# Network analysis of the left anterior descending coronary arteries in swim-trained rats by an in situ video microscopic technique

**DOI:** 10.1186/s13293-021-00379-y

**Published:** 2021-05-26

**Authors:** Marianna Török, Petra Merkely, Anna Monori-Kiss, Eszter Mária Horváth, Réka Eszter Sziva, Borbála Péterffy, Attila Jósvai, Alex Ali Sayour, Attila Oláh, Tamás Radovits, Béla Merkely, Nándor Ács, György László Nádasy, Szabolcs Várbíró

**Affiliations:** 1 Department of Obstetrics and Gynecology, Semmelweis University, Üllői u. 78/a, Budapest, 1082 Hungary; 2 Institute of Clinical Experimental Research, Semmelweis University, Tűzoltó u. 37-47, Budapest, 1094 Hungary; 3 Department of Physiology, Semmelweis University, Tűzoltó u. 37-47, Budapest, 1094 Hungary; 4 Department of Neurosurgery, Military Hospital, Róbert Károly körút 44, Budapest, 1134 Hungary; 5 Heart and Vascular Center, Semmelweis University, Városmajor u. 68, Budapest, 1122 Hungary

**Keywords:** Ventricular hypertrophy, Exercise training, Resistance coronary arteries, Network, Sex differences, Video microscopic technique

## Abstract

**Background:**

We aimed to identify sex differences in the network properties and to recognize the geometric alteration effects of long-term swim training in a rat model of exercise-induced left ventricular (LV) hypertrophy.

**Methods:**

Thirty-eight Wistar rats were divided into four groups: male sedentary, female sedentary, male exercised and female exercised. After training sessions, LV morphology and function were checked by echocardiography. The geometry of the left coronary artery system was analysed on pressure-perfused, microsurgically prepared resistance artery networks using in situ video microscopy. All segments over > 80 μm in diameter were studied using divided 50-μm-long cylindrical ring units of the networks. Oxidative-nitrative (O-N) stress markers, adenosine A_2A_ and estrogen receptor (ER) were investigated by immunohistochemistry.

**Results:**

The LV mass index, ejection fraction and fractional shortening significantly increased in exercised animals. We found substantial sex differences in the coronary network in the control groups and in the swim-trained animals. Ring frequency spectra were significantly different between male and female animals in both the sedentary and trained groups. The thickness of the wall was higher in males as a result of training. There were elevations in the populations of 200- and 400-μm vessel units in males; the thinner ones developed farther and the thicker ones closer to the orifice. In females, a new population of 200- to 250-μm vessels appeared unusually close to the orifice.

**Conclusions:**

Physical activity and LV hypertrophy were accompanied by a remodelling of coronary resistance artery network geometry that was different in both sexes.

## Background

The network geometry of the coronary arteries is very important in determining blood flow, but the methodical techniques for examining geometric properties are very difficult and expensive. Computed tomographic coronary angiography (CTCA) or coronary magnetic resonance (MR) angiography are possible examination methods [1–5]; 3D reconstruction is even possible [3]. However, only larger conduit arteries can be examined with these methods. There have been a limited number of publications in the literature studying alterations in small coronary artery networks [6–9]. Whole coronary artery network analysis (both conduit and resistance arteries) and comparisons with these methods are very complicated because each coronary network has a unique pattern. Previously, network remodeling of intramural coronary resistance arteries in aged rats and in hypertensive female rats was analysed with micropreparation and in situ video microscopic recording by Nádasy et al. [6, 7]. In aged rats and in rats with angiotensin infusion-induced hypertension, network rebuilding occurred that unfavourably changed hemodynamic resistance, including increased segmental tortuosity, parallel running branches, broken courses of larger branches, and multiple branchings and branch crossings [6, 7]. In the present study, the questions of whether the network geometry adapted as a result of long-term exercise training and whether there are any differences in long-term exercise-induced coronary resistance artery network remodeling between the two sexes were addressed.

There is increasing evidence that an active lifestyle decreases the risk of coronary artery disease [10]. Such effects are induced by systemic metabolic factors [11], by reducing pathological alterations in large coronary vessels [12] and by improving ventricular microcirculation [13]. Furthermore, regular physical activity reduces obesity and helps keep the body weight at an optimal level [14]. Long-term, regular exercise induces a positive remodeling of the coronary resistance arteries themselves [15, 16]. There are substantial differences in the structure and function of male and female coronary resistance arteries [17], and we can suppose that the effects of long-term exercise training on small coronary arteries and arterioles will be different in the two sexes. As a result of regular physical activity, the walls of large (conduit) coronary arteries and skeletal muscle arteries become thinner, and the lumens expand [15, 18], whereas the walls of the smaller resistance coronary arteries become thicker and more muscular [16]. Exercise training changes not only the morphology of the coronary arteries but also their function. The endothelial dilatation capacities of large and small coronary arteries are enhanced after exercise [18–20]. Endothelium-independent (adenosine) relaxation increases in conduit and large-resistance coronary arteries as a result of exercise training, but it has been found to be unaltered in small-resistance coronary arteries of trained rats [21]. Basal tone is higher in moderately trained coronary resistance arteries, which elevates the potential range of vasomotion during acute physical exercise. The balance between vasodilator and vasoconstrictor endogenous prostanoids favourably changes [16]. Substantial sex differences in endothelial relaxation have also been demonstrated with training in other vessels: the flow-mediated dilatation of the brachial artery increases in men but not in women after acute exercise [22–24]. Furthermore, the myogenic tone and the agonist-induced contraction (thromboxane agonist) of coronary arteries are greater only in female rats after strenuous physical exercise [25].

Oxidative stress reflects the imbalance between reactive oxygen radical production and adequate antioxidant protection. This is an unfavorable condition that can lead to cell and tissue damage and plays a role in aging; inflammatory processes; the development, initiation, and progression of cardiovascular, neurodegenerative, and cancerous lesions [26, 27]. The relationship between exercise and oxidative stress is extremely complex, depending on the type, intensity and length of the workout [28, 29]. In addition to the many positive health effects of regular, moderate exercise, it also reduces oxidative stress. In contrast, acute training leads to increased oxidative stress, although this stimulus is required to activate endogenous antioxidant protection and thus indirectly to reduce chronic oxidative stress [30].

Oxidative and nitrative stress markers include 4-hidroxy-2 nonenal (HNE), poly(ADP)-ribose (PAR), 3-nitrotyrosine (NT) and endothelial nitric oxide synthase (eNOS) [31–35]. The formation of HNE-protein-adducts is one of the accompanying processes in oxidative stress [31]. The level of PAR polymer can be considered as a molecular sensor of DNA damage, its level is significantly increased in diseases with oxidative and nitrative stress, such as inflammation or diabetes mellitus [32, 33]. 3-Nitrotyrosine is considered a relatively specific marker of peroxynitrite-mediated damage [34, 35]; levels are significantly elevated in many disease processes such as asthma, stroke or multiple sclerosis [35]. eNOS not only has antihypertensive, antithrombotic and antiatherosclerotic effects [36], but may be an important marker of oxidative stress. The superoxide anions react with the nitric oxide produced by eNOS to form peroxynitrite, which severely damages proteins, lipids and DNA, leading to cell damage and apoptosis [37].

Adenosine is an endogenous nucleoside which plays an important role in physiological and pathophysiological conditions within the coronary network. It acts primarily on the A_2A_ receptor (Ade A_2A_-R). In coronary heart disease, it is released from the heart muscle, endothelium, and immune cells during hypoxia, ischemia, or inflammation. A_2A_ receptor activation improves coronary circulation and has an anti-inflammatory effect [38].

Vascular endothelial growth factor (VEGF) plays a key role in angiogenesis associated with regular physical activity. VEGF is responsible for coordinating among mechanical, chemical and transcriptional mediators as a result of exercise [39]. Previous studies have shown that there is an immediate increase in mRNA of VEGF upon exercise in skeletal and cardiac muscles [39]. The increase in VEGF mRNA levels is closely related to the function of hypoxia-induced factor 1-alpha (HIF-1-α). During exercise, tissue oxygen levels decrease to an extent already sufficient for HIF-1-α activation, leading to VEGF activation [39]. The endothelial nitric oxide synthase (eNOS) plays a key role in the cardiovascular protective effect of exercise. Genetic deletion [40] or pharmacological inhibition [41] eliminate its cardioprotective effect. In the heart of the rodent, eNOS is located in 80% of the coronary endothelium [42].

Estrogen may play an important role in exercise-induced remodeling. Of its two receptors (α and β), β receptor activation plays a role in angiogenesis in many tissues and improves endothelial dilatation [43, 44].

In the present study, we show how the intramural resistance coronary artery network running underneath the surface of the rat heart is altered after 12 weeks of swim training and what differences can be detected between males and females.

## Materials and methods

### Ethical approval and animals

Young adult, 50- to 54-day-old age-matched male (*n* = 18) and female (*n* = 20) Wistar rats were included in the coronary network preparation study. Rats were housed in standard cages at constant room temperature (22 ± 2 °C) and humidity with 12-h light–dark cycles. They were supplied ad libitum with standard laboratory rat diet and with tap water. Experimental plans were approved by the Animal Care Committee of Semmelweis University and Hungarian authorities (Permission Number: PEI/001/2374-4/2015). Throughout the experiments, the regulations of the ‘Guide for the Care and Use of Laboratory Animals’ by the National Institutes of Health (NIH Publication No. 86-23, revised 1996.) and the EU Directive 2010/63/EU were followed.

### Chemicals

Pentobarbital (Euthasol, CEVA Santé Animale, Libourne, France) was used for anaesthesia. For in vitro studies, we used a normal Krebs-Ringer solution (nKR), which consisted of the following (in mmol/l): 119 NaCl, 4.7 KCl, 1.2 NaH_2_PO_4_, 1.17 MgSO_4_, 24 NaHCO_3_, 2.5 CaCl_2_, 5.5 glucose and 0.0345 EDTA. Chemicals were purchased from Reanal (Budapest, Hungary).

### Experimental groups and swim training protocol

After 7 days of acclimatization, animals were randomly divided into one of the following four experimental groups: male sedentary (MSed, *n* = 8), male exercised (MEx, *n* = 10), female sedentary (FSed, *n* = 10) and female exercised (FEx, *n* = 10). The numbers of animals for these groups were 8, 10, 10 and 10, respectively. Exercised male and female rats were subjected to a strenuous long-term swim training programme. After initial adaptation, both male and female rats swam for a total period of 12 weeks, for 200 min/day, 5 days a week as previously described [45]. Briefly, animals were placed in a water tank (with plain walls) divided into 6 lanes filled with tap water (30–32 °C) at the same time of day in all training sessions. For adequate adaptation, the initial swimming duration was 15 min on the first day, and the duration of swimming was increased by 15 min every 2 days until reaching the maximal 200 min/day. Untrained control male and female Wistar rats were placed in the water tank for 5 min each day during the 12-week swim training programme.

### Echocardiography

Echocardiography was performed at the end of the experimental period as described previously [46]. In brief, rats were anaesthetized with 1–2% isoflurane in 100% oxygen. Animals’ body temperature was maintained at 37 °C with a heating pad. The transthoracic echocardiography was conducted by using a 13-MHz linear transducer (GE, Healthcare, Horten, Norway) attached to a Vividi Echocardiac Image Analysis System (GE, Healthcare, USA). Mid-papillary-level short-axis B-mode images were used to measure left ventricular (LV) anterior and posterior wall thicknesses (AWT and PWT, respectively) in diastole and LV end-diastolic and end-systolic diameters (LVEDD and LVESD, respectively). Images were analysed by blinded investigators with image analysis software (EchoPac v113, GE, Healthcare). The values of the three consecutive cycles were averaged and used for statistical analysis. The computed parameters were as follows: LV mass = [[(LVEDD + AWTd + PWTd)^3^–LVEDD^3^] × 1.04] × 0.8 + 0.14 standardized for body mass, (LV mass index); fractional shortening (FS) = (LVEDD − LVESD)/LVEDD × 100]. The ventricular volume values and ejection fraction (EF) were computed according to the Teichholz method ((EF) (%) = (LVEDV − LVESV)/LVEDV × 100 [47]).

### Micropreparation and in situ video microscopic recording of whole coronary networks

At the end of the swim training programme, blood pressure was measured via the cannulation of the right carotid artery (Gould pressure heads) under pentobarbital anaesthesia (45 mg/kg body weight, intraperitoneal). Then, animals were perfused with 150 ml of nKR solution to remove all blood from the vessels. The heart was removed to prepare the left anterior descending (LAD) coronary artery network. For the network preparation, we used a Wild M3Z preparation microscope as described previously [7, 48]. In brief, the heart was pinned down in a rubber-bottomed Petri dish and covered with cooled normal Krebs solution. By careful microsurgical preparation, the whole intramural network of the LAD was prepared and left in situ to maintain the original branching geometry. Larger branches of the LAD mostly run in the ventricular muscle tissue, parallel with the surface, a few hundred micrometres deep. Preparation followed the small arteries and large arterioles toward the periphery to vessels with diameters of approximately 80 μm. Then, the orifice was cannulated with plastic cannulas (400 μm), and the network was perfused with Krebs-Ringer solution at close to in vivo pressures. Both the perfusate and superfusate nKR solutions were kept at constant temperatures of 37 °C and bubbled with a gas mixture of 5% CO_2_, 20% O_2_ and 75% N_2_, which stabilized the pH at 7.4. After 2 min of equilibration, without any added vasoactive substance, the LAD network was recorded by a video microscope using low and high magnifications (8.58 and 1.47 μm/pixel, respectively). The optical angle of the video microscope tube was kept perpendicular with the photographed surface, vertical, and bent approximately 30° to the left, right, apically and toward the root of the heart to photograph the appropriate sections of the network. The analysis of the network pictures was performed off-line with the help of specific image-analyzing software (ImageJ, NIH, Bethesda, MA, USA). The length calibration was made with a micrometre etalon (Wild, Heerbrugg, Switzerland).

### Geometric analysis

#### High-magnification reconstruction of the network

**Fig. 1:**
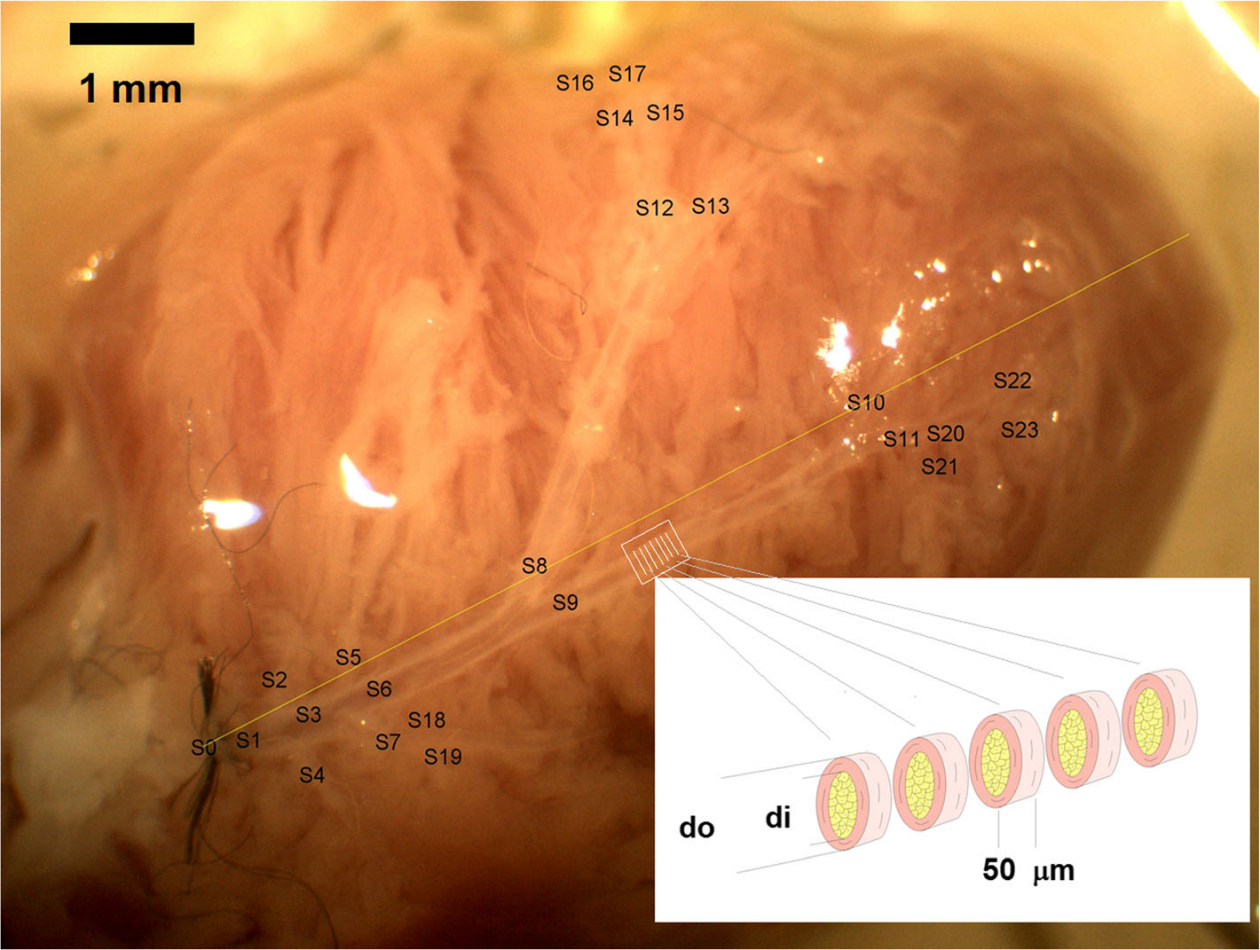
Microprepared, pressure-perfused, video microscopy images of intramural coronary resistance artery network branches of the left coronary artery of an exercised male rat. Note the orifice–apex axis drawn and the numbering of segments. Scale bar, 1 mm

Using the low-magnification pictures, high-magnification pictures were selected with good visibility and perpendicular position of the parts of the network with the microscope tube axis. The picture of the whole network was then reconstructed in the form of a collage of high-magnification pictures. Further measurements were made on this reconstructed network, which contained all larger branches of the LAD stretched in the horizontal plane. Segments of the network were then numbered (Fig. 1).

#### Analysis of bifurcations (branchings)

All bifurcations (branchings) in the > 80-μm range were identified (361 in the four groups) and were characterized by the inner diameters of the mother and daughter branches as well as by the angles of the axis of daughter branches with that of the mother branch.

#### Vascular ring unit analysis

The networks were divided (theoretically) into 50-μm-long ring units. A Cartesian coordinate system was laid upon the high-magnification collage picture of the network. The *X*-axis was drawn between the lower contour of the orifice and the apex [7]. The *Y*-axis was drawn perpendicularly with it through the orifice, with positive values toward the left ventricle. The orifice was defined as the zero point. For each 50-μm-long ring unit, with several hundreds of them for each network, the outer and inner radii, wall thickness, *X* and *Y* coordinates for the ring centre, angle of axis with the *X*-axis, direct distance from the orifice and flow distance along the flow route from the orifice were computed. The overall number of ring units in the four groups, 38 animals, was 29,390.

#### Mathematical analysis of alterations in network geometry

**Fig. 2:**
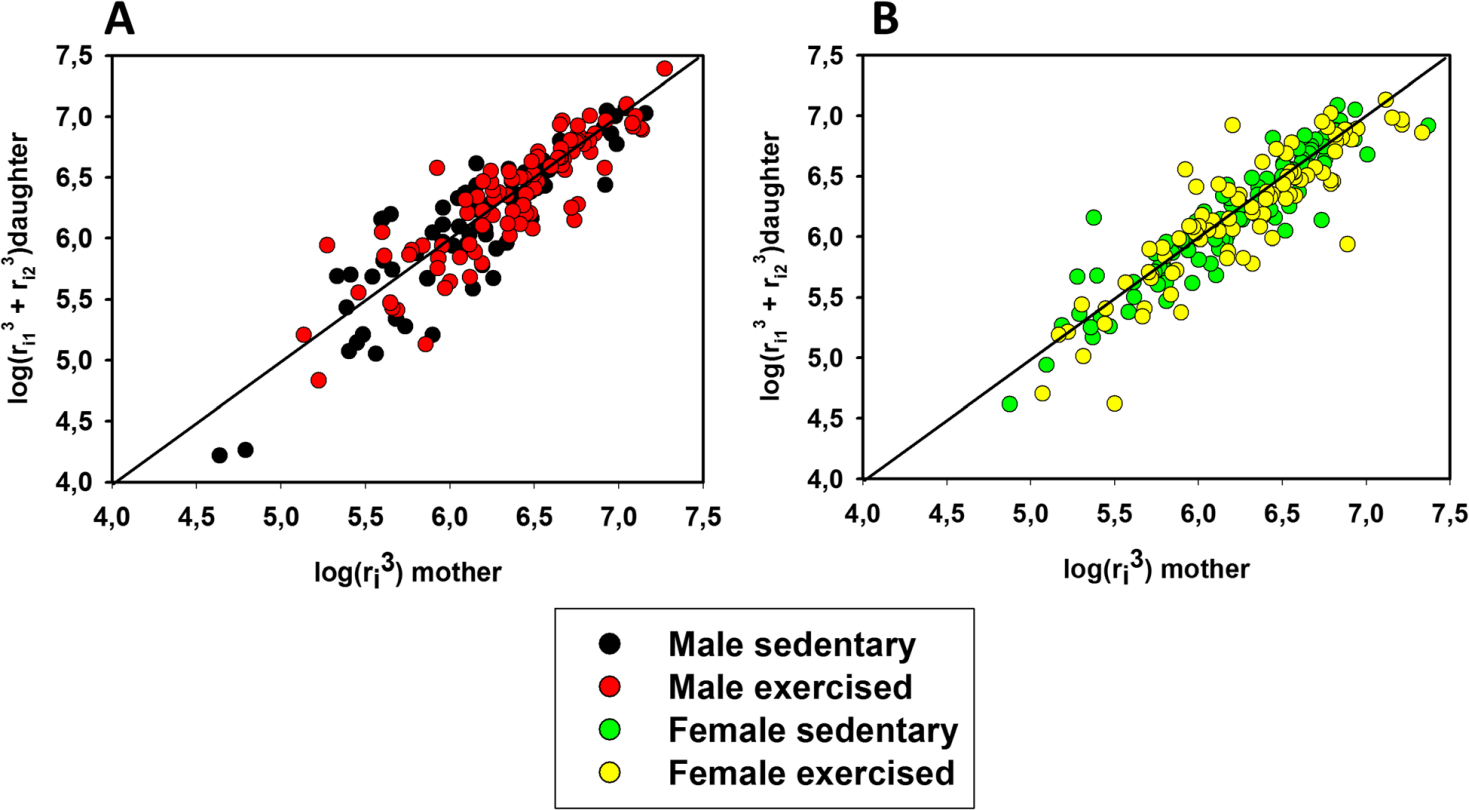
Analysis of bifurcations (branchings). The validity of Murray’s law in the left anterior descending coronary resistance artery network was checked. The sum of the cube of the lumen radii of daughter branches was plotted against the cube of the lumen radius of the mother branch in logarithmic scale. **a** Control and exercised males; **b** control and exercised females. The line connects points where Murray’s law is valid. Note substantial adherence to the line in all four groups. Deviations from the theoretical value were not significantly different in any of the comparisons

**Fig. 3:**
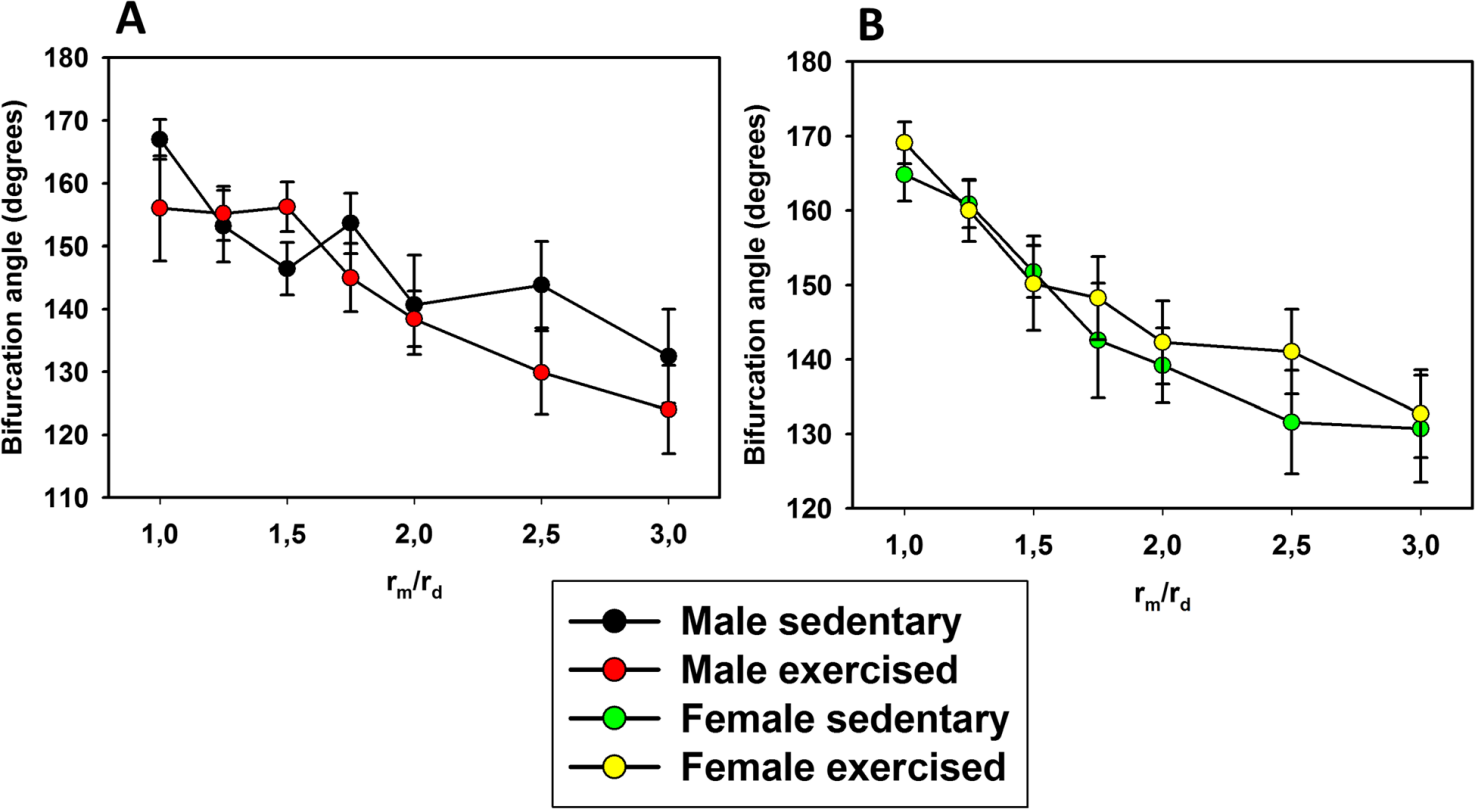
Analysis of bifurcations (branchings). Angle of the axis of the daughter branch with the axis of the mother branch as a function of the ratio of lumen radii (*r*_imother_/*r*_idaughter_). **a** Control and exercised males; **b** control and exercised females. Note that smaller daughter branches tend to deviate more from the course of the mother branch (angles closer to the perpendicular 90°), while larger daughter branches tend to follow the course of that of the mother branch (approximately 180°). Pearson correlations were significant in all four groups, with no significant differences between groups

**Fig. 4:**
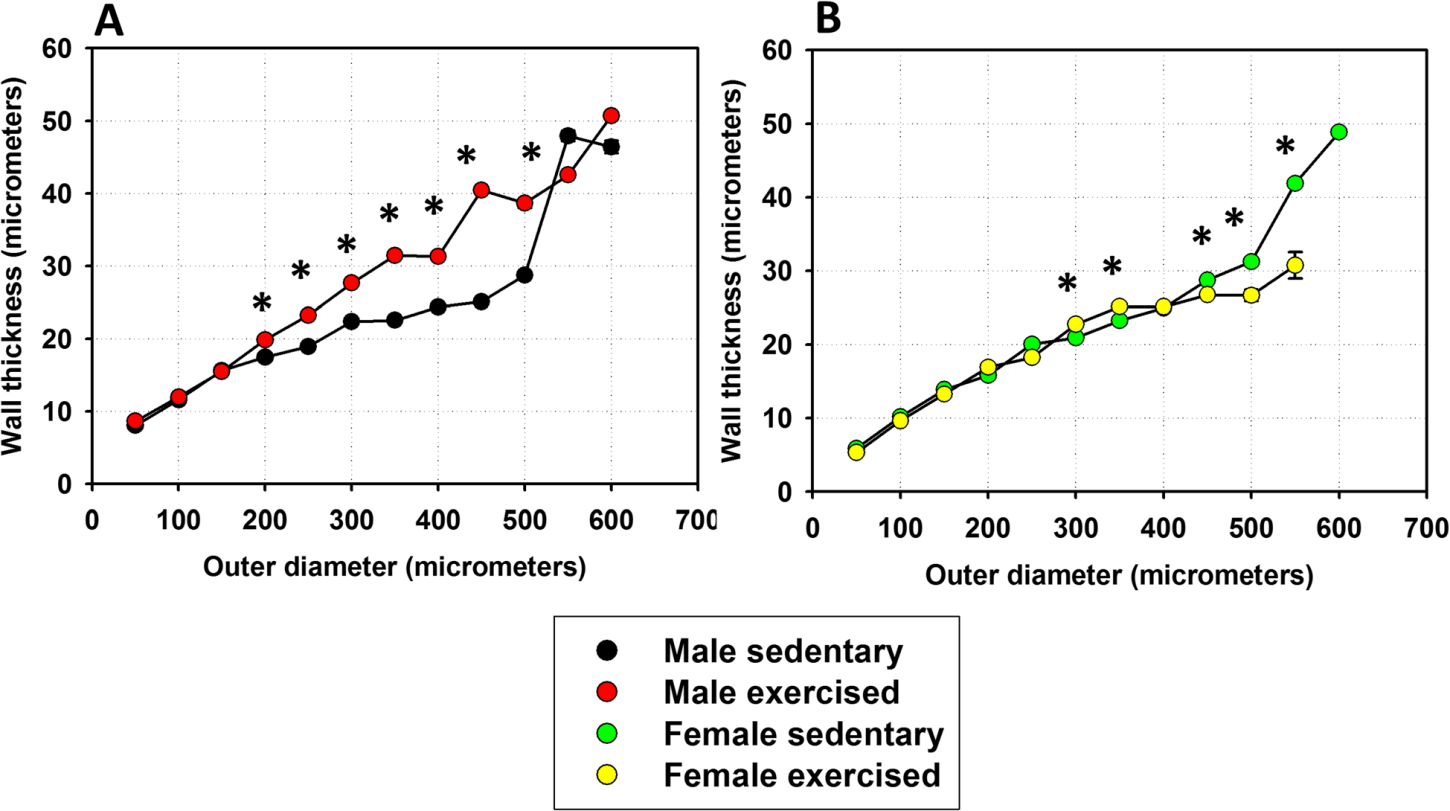
Analysis of ring elements of the whole left anterior descending coronary artery system. Wall thickness as a function of outer diameter. **a** Control and exercised males and **b** control and exercised females. Wall thickness positively correlated with diameter in all four groups. Significance levels of paired Tukey’s comparisons are shown, ^*^ *p* < 0.05 between sedentary and trained. Note increased wall thickness in trained male animals, while in females, a slight but significant wall thickness reduction could be observed. Female vessels had significantly thinner walls than males in the 100- to 300-μm range in the sedentary groups and in the whole range of the trained groups (not shown on the figure). (Two-way ANOVA with post hoc Tukey’s test. **a** In the 200–500-μm range, dF = 1 and 6; *F* = 1090.1, *p* < 0.001. Exercise effect is dependent on vessel diameter (interaction, *p* < 0.001). **b** In the 300–350-μm range, dF = 1 and 5, *F* = 59.5, *p* < 0.001. Exercise effect is dependent on vessel diameter (interaction, *p* < 0.001))

**Fig. 5:**
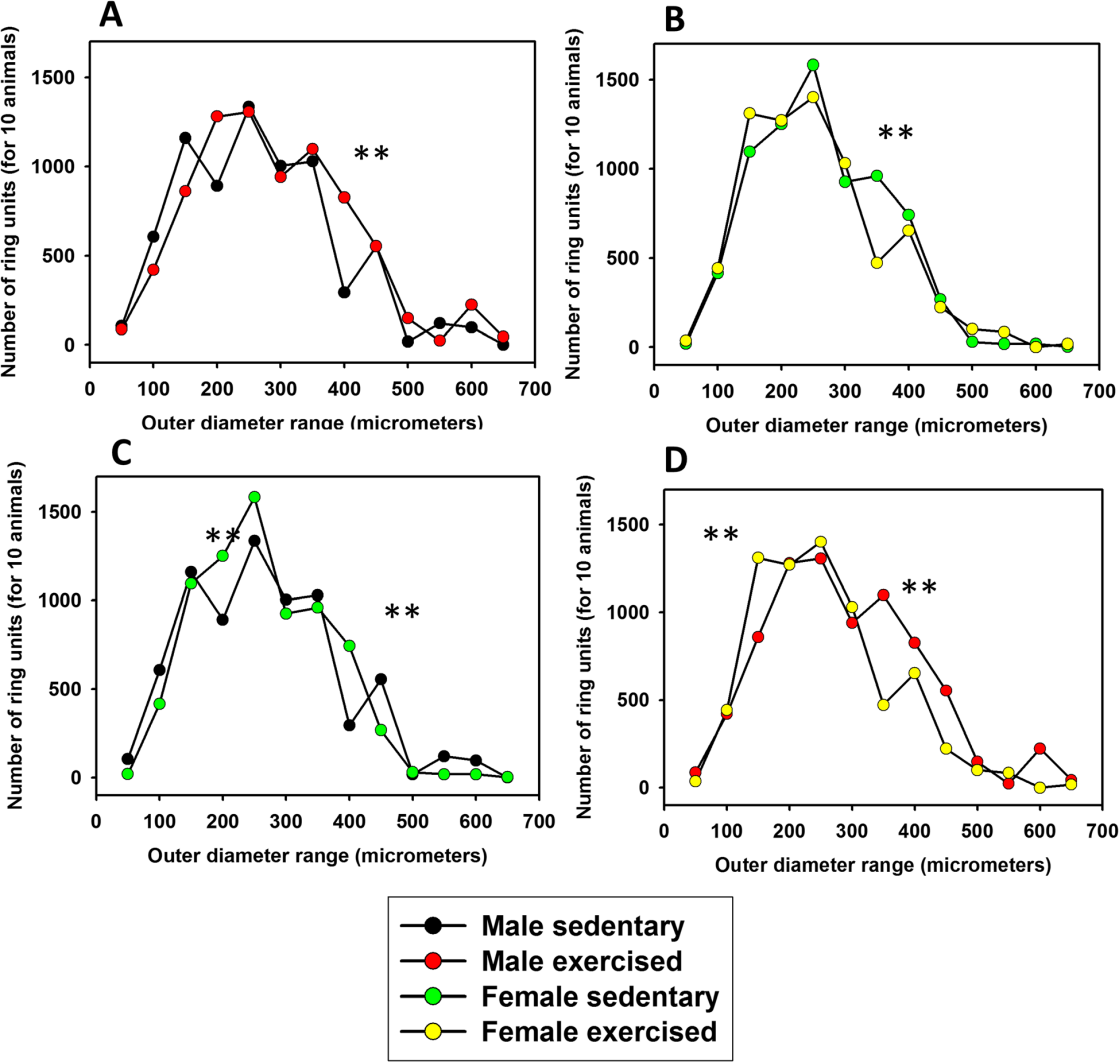
Ring unit analysis. Numbers of 50-μm-length ring units in the networks in different outer diameter ranges. Pooled data for 10 animals, with a total of 29,390 ring units included. **a** Control and exercised males; **b** control and exercised females; **c** control males and females; **d** exercised males and females. Ring frequency spectra of trained animals were significantly different from the sedentary ones in both males and females (^**^ *p* < 0.01 with the *χ*^2^ probe). Note doubling of the 400-μm vessel units and substantial elevation of 200-μm vessel units in males in response to strenuous training. In females, the number of 150-μm vessel units was elevated, while there was a reduction in the 350-μm range. The ring frequency spectra of male animals were significantly different from the female ones in both the sedentary and exercised groups (*p* < 0.001 with the *χ*^2^ probe). In the control groups, the numbers of the larger (300–600 μm) vessels are larger, and the numbers of the smaller (200–250 μm) ring units are smaller in the male rats than in the female rats. In the exercised groups, there are more 350- to 600-μm vessels and fewer 200- to 250-μm vessels in male animals than in female animals. (**a** Chi-square = 646.858 with 12 degrees of freedom, *p* < 0.001. **b** Chi-square = 322.665 with 12 degrees of freedom, *p* < 0.001. **c** Chi-square = 613.675 with 12 degrees of freedom, *p* < 0.001. **d** Chi-square = 773.079 with 12 degrees of freedom, *p* < 0.001)

**Fig. 6:**
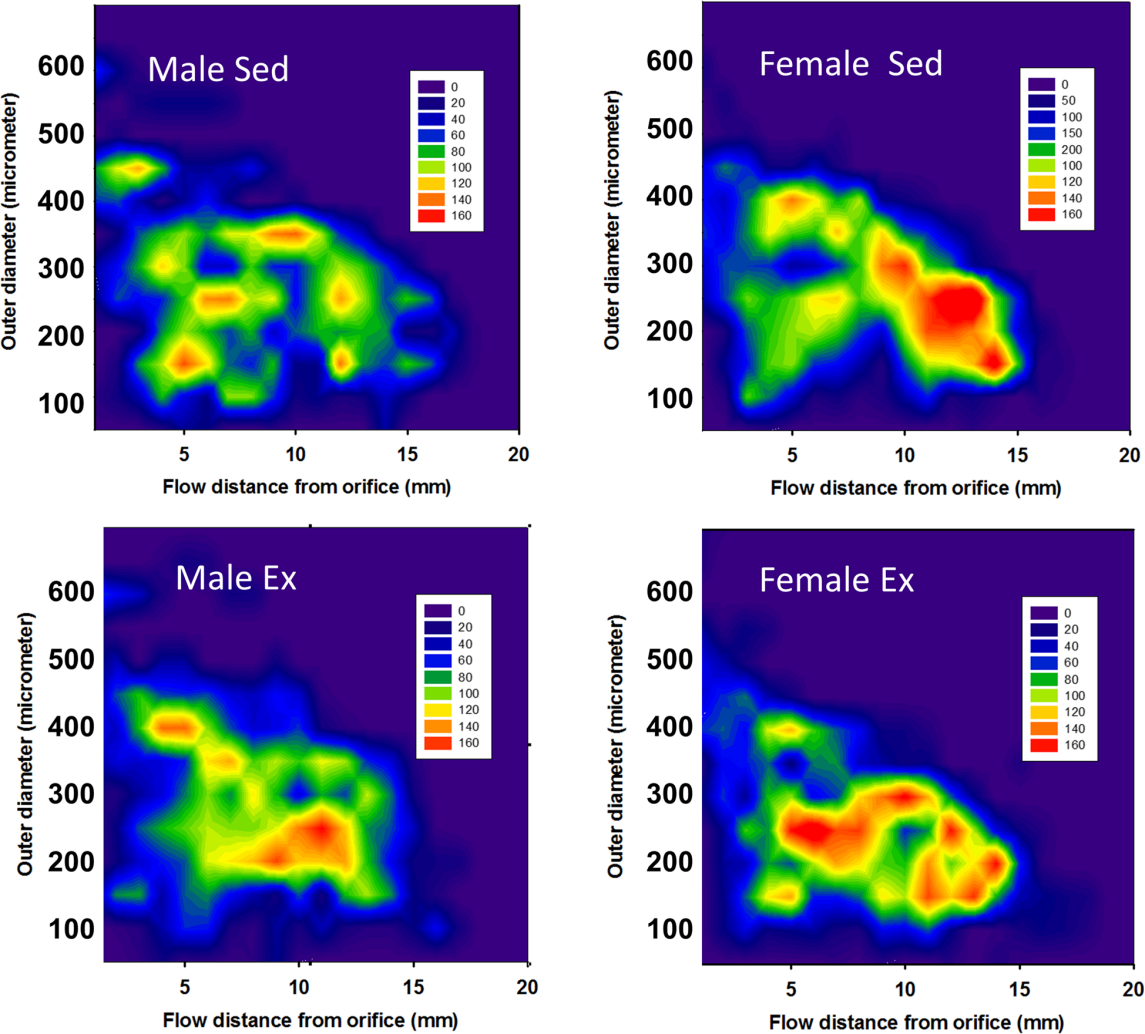
Distribution of different vascular elements in the network. Bidimensional histograms of ring unit frequencies according to outer diameter and flow distance from the orifice. In the case of males, note that the increased number of 200-μm elements after training seems to be the result of the morphological dilation of 100- to 150-μm elements in a fair distance from the orifice. Clusters of 400-μm elements appear close to the orifice. This way, a younger network is formed (negative correlation between diameter and flow distance). In the case of females, the network in sedentary animals is fairly well organized, while in the strenuous exercise network, 150- to 200-μm vessel elements appear close to the orifice, not improving but loosening the pattern

Potential alterations in network geometry and sex differences were analysed as follows: Branchings were tested for the validity of Murray’s law. According to the law, the sum of the cubes of the lumens of the daughter branches is equal to the cube of the lumen of the mother branch (Murray), which ensures identical shear rates induced by blood flow at the endothelium (Fig. 2). Characteristics of branching angles were tested on plots on which the angle of the axis of the daughter branch with the axis of the mother branch was plotted against the ratio of mother/daughter lumina. The value is 90° where the daughter branch arises perpendicularly and 180 degrees if it continues the course of that of the mother branch (Fig. 3). Remodeling processes often alter lumen diameter/wall thickness ratios. Such plots are shown in Fig. 4. In previous works, we found it fruitful to construct histograms of the frequency of ring units in separate diameter ranges, which can reveal remodelling processes of resistance artery populations (Fig. 5). Such histograms yield even more detailed insight if two-dimensional histograms are constructed—one dimension being the lumen diameter range, and the other being the distance through which the blood reaches the ring unit in question (computed from the orifice) [6, 7] (Fig. 6).

### Immunohistochemical examinations

The whole heart was placed in 4% formaldehyde for fixation (*n* = 4–4). After dehydration, the tissues were embedded in paraffin and were cut into 5-μm-thick sections. Immunohistochemistry was performed against oxidative and nitrative stress markers, such as 4-hydroxy-2-nonenal (HNE), poly(ADP)-ribose (PAR), 3-nitrotyrosine (NT), endothelial nitric oxide synthase (eNOS) as well as for vascular endothelial growth factor receptor 1 (VEGFR-1), adenosine A_2A_-R (AdeA_2A_-R) and estrogen receptor (ER). Polyclonal rabbit Anti-HNE (1:500, Abcam, ab46545, Cambridge, MA, USA), Anti-NT (1:500, Abcam, ab42789, Cambridge, MA, USA), Anti-VEGFR-1 (1:50, Novus Biologicals, VEGF-R1/Flit-1 Antibody NB100-92237, Centennial, CO, USA), Anti-ERα (1:100, Merck/Sigma-Aldrich, #06-935, Budapest, Hungary) and monoclonal mouse Anti-PAR (1:1000, Tulip Biolabs., Cat. #1020, Landsdale, PA, USA), Anti-eNOS (1:50, Abcam, ab76198, Cambridge, MA, USA) and Anti-AdeA_2A_-R (1:50, Santa Cruz Biotechnology INC, 7F6-G5-A2, sc-32261, Dallas, TX, USA) antibodies were applied. Secondary labeling was achieved by using horseradish-peroxidase (HRP)-labeled horse anti-rabbit or anti-mouse IgG polymer detection kit (Vector Laboratories, MP-7401, MP-7402, Burliname, CA, USA, 30 min). Visualization of specific labeling with brown-colored 3-3’-diamino-benzidine (DAB) peroxidase HRP substrate kit (Vector Laboratories, SK-4100, Burlingame, CA, USA) diaminobenzidine (DAB) as colored substrate and hematoxylin counterstaining was achieved by blue-colored hematoxylin QS nucleus stain (Vector Laboratories, H-3404-100, Burlingame, CA, USA). Microscopic images of immunostained tissue sections were taken by microscope-coupled videocamera (Nikon Eclipse NI NI-SS, 933584 with Nikon DS-Ri2 camera and NIS Elements BR image software, Nikon Corporation, Japan). Non-calibrated optical density and staining intensity of specific staining was measured by ImageJ software (NIH, Bethesda, MA, USA).

### Statistical evaluation

For statistical analysis, SPSS Sigma Stat software and Excel functions were used. All data are presented as the mean ± SEM. The normal distribution was checked with the Shapiro-Wilk test. Two-way analysis of variance (ANOVA) was used with the post hoc Tukey test for paired comparisons, and the level of significance was set at *p* < 0.05. Ring unit diameter distribution histograms of pooled data were compared with the *χ*^2^ test.

## Results

### Physiological alterations

As a result of the swim training programme, body weight was significantly reduced in male animals compared to control animals despite ad libitum feeding (Table 1). Body weights significantly differed between the male and female groups in both control and exercised animals (Table 1). The calculated LV mass index determined by echocardiography indicated cardiac hypertrophy in both exercised groups compared with control animals (Table 1). The LV mass index was higher in female rats than in males in both the control and exercised groups, and female sex was associated with greater relative heart hypertrophy (Table 1). The systolic function of the hypertrophied heart (determined by the ejection fraction and fractional shortening) was significantly increased in exercised rats compared with sedentary animals in both the male and female groups (Table 1). The EF and FS were greater in female animals than in male rats in control groups, whereas they were not different between the trained groups (Table 1). The mean arterial pressure did not differ between groups (Table 1).

**Table 1:** Basic characteristics and hemodynamic parameters in the study groups

	MSed	MEx	FSed	FEx	p_**i**_
BW, g	487 ± 11	424 ± 9^*^	287 ± 4^*^	**286 ± 4**^**§**^	**< 0.001**
LV mass index, g/kg	2.36 ± 0.08	3.05 ± 0.08^*^	3.07 ± 0.08^*^	**3.64 ± 0.10**^**#§**^	0.471
EF, %	73 ± 1.0	81 ± 1.2^*^	79 ± 0.8^*^	**82 ± 0.9**^**#**^	**0.017**
FS, %	44 ± 0.9	52 ± 1.3^*^	49 ± 0.8^*^	**52 ± 1.1**^**#**^	**0.035**
MAP, mmHg	124 ± 9	123 ± 8	105 ± 7	110 ± 5	0.087

*BW* body weight at the end of the training program. (*F*_training_ = 15.193, *F*_sex_ = 627.244, *F*_int_ = 13.216, df_training_ = 1, df_sex_ = 1, df_int_ = 1, *P*_training_ < 0.001, *P*_sex_ < 0.001 and *P*_int_ < 0.001); LV mass index, left ventricular mass standardized for body mass. (*F*_training_ = 64.55, *F*_sex_ = 70.00, *F*_int_ = 0.53, df_training_ = 1, df_sex_ = 1, df_int_ = 1, *P*_training_ < 0.001, *P*_sex_ < 0.001 and *P*_int_ = 0.47); *EF* ejection fraction. (*F*_training_ = 29.44, *F*_sex_ = 10.36, *F*_int_ = 6.26, df_training_ = 1, df_sex_ = 1, df_int_ = 1, *P*_training_ < 0.001, *P*_sex_ = 0.003 and *P*_int_ = 0.02); *FS* fractional shortening. (*F*_training_ = 28.33, *F*_sex_ = 6.43, *F*_int_ = 4.82, df_training_ = 1, df_sex_ = 1, df_int_ = 1, *P*_training_ < 0.001, *P*_sex_ = 0.02 and *P*_int_ = 0.04); *MAP* mean arterial pressure. (*F*_training_ = 0.008, *F*_sex_ = 5.995, *F*_int_ = 0.02, df_training_ = 1, df_sex_ = 1, df_int_ = 1, *P*_training_ = 0.93, *P*_sex_ = 0.019 and *P*_int_ = 0.865)

^*^ *p* < 0.05 vs. MSed, § *p* < 0.05 vs. MEx, # *p* < 0.05 vs. FSed

### Bifurcations (branchings)

Lumen diameters of bifurcation adhered fairly well to Murray’s law: the sum of the cubes of the lumen radii of the daughter branches was close to the cube of the mother branch. Deviations from theoretical values did not differ in the four groups in any of the comparisons (Fig. 2). In a similar manner, branching angles (defined as the angle between the axis of the mother branch and that of the daughter branch) increased with increasing lumen of the daughter branch. Negative correlations between the angles of axes and the ratios of diameters (*r*_*m*_/*r*_*d*_) were equally characteristic for the four groups (Fig. 3).

### Ring unit analysis

There were positive correlations of wall thickness with outer diameter in all four groups (Fig. 4). There was a significant elevation of wall thickness of the resistance arteries in the case of exercised male animals, while in females, a statistically significant reduction in wall thickness occurred in the larger arteries (Fig. 4). Females had slightly but significantly thinner walls than males in the sedentary state, but in exercised animals, female coronary vessels were significantly and massively thinner than those of males in the whole diameter range (not shown directly on the figure).

Ring frequency histograms (number of 50-μm ring units, pooled for 10 animals) were significantly different between trained and sedentary animals in both sexes (*p* < 0.01 with the χ^2^ probe, Fig. 5). In swim-trained males, the number of 400-μm rings almost doubled, and there was a substantial elevation in the number of ring units also in the 200-μm range compared with sedentary ones (Fig. 5a). In the case of females, the number of 150-μm rings increased, while there was a reduction in the number of 350-μm units (Fig. 5b). The numbers of ring units did not differ in the four groups (718 ± 68, 780 ± 47, 730 ± 45 and 705 ± 30 ring unit elements per heart for the MSed, MEx, FSed and FEx groups, respectively, n.s.). Ring frequency spectra were significantly different between male and female animals in both the sedentary and trained groups (*p* < 0.01 with the *χ*^2^ probe). Sedentary male networks had less abundant 200- to 250- and 400-μm vascular units and more abundant 350- and > 500-μm units (Fig. 5c). Trained males had more abundant 350- to 450-μm units and less abundant 150-μm diameter elements than corresponding trained females (Fig. 5d).

### Network distribution

The next question was at what points of the network these characteristic differences in the frequency of vascular units appear. To answer this question, two-dimensional ring unit frequency histograms were constructed, with one axis showing the outer diameter and the other showing the distance from the orifice following the route of blood flow with the number of units colour-coded (Fig. 6). Figure 6a and c demonstrate that the elevation of the number of thicker 400-μm units occurred at the ‘right’ place, close (3–5 mm) to the orifice, while population extension of 200-μm units occurred again where it was advantageous, at an 8- to 13-mm distance from the orifice. For females in the sedentary state, a cluster of 150- to 350-μm units at a distance of 10–15 mm is characteristic with the appearance of a new 150- to 250-μm cluster at a close flow distance (5–8 mm) from the orifice after exercise (Fig. 6b and d).

In the present study, we show how the intramural resistance coronary artery network running underneath the surface of the rat heart is altered after 12 weeks of swim training and what differences can be detected between males and females.

### Immunohistochemical examinations

**Fig. 7:**
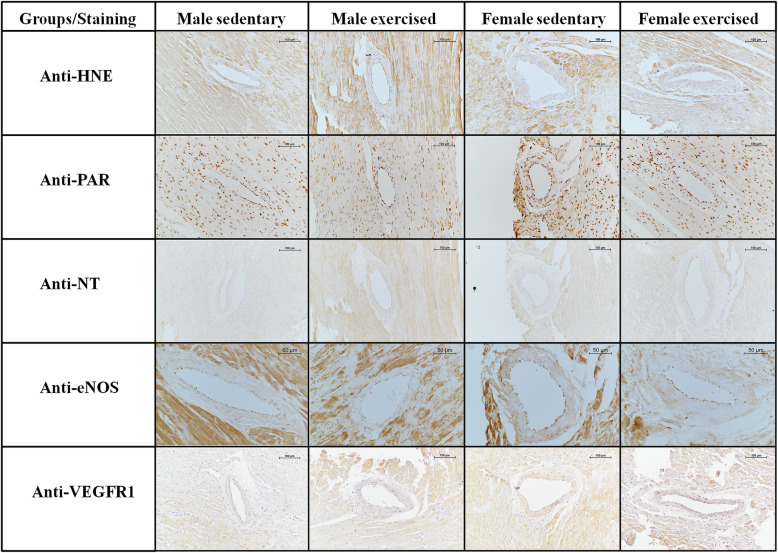
Representative photos of 4-hydroxy-2-nonenal (HNE), poly(ADP)-ribose (PAR), 3-nitrotyrosine (NT), endothelial nitric oxide synthase (eNOS) and vascular growth factor receptor 1 (VEGFR-1) immunohistochemistry sections of control and exercised male and female coronary segments. Scale bar 50 or 100 μm. (Two-way ANOVA with post hoc Tukey’s test. Anti-HNE, *F*_training_ = 0.375, *F*_sex_ = 0.00135, *F*_int_ = 0.000644, df_training_ = 1, df_sex_ = 1, df_int_ = 1, *P*_training_ = 0.553, *P*_sex_ = 0.971 and *P*_int_ = 0.98; Anti-PAR, *F*_training_ = 0.0136, *F*_sex_ = 5.128, *F*_int_ = 0.00267, df_training_ = 1, df_sex_ = 1, df_int_ = 1, *P*_training_ = 0.909, *P*_sex_ = 0.04 and *P*_int_ = 0.873; Anti-NT, *F*_training_ = 2.849, *F*_sex_ = 0.0874, *F*_int_ = 0.000248, df_training_ = 1, df_sex_ = 1, df_int_ = 1, *P*_training_ = 0.114, *P*_sex_ = 0.772 and *P*_int_ = 0.988; Anti-eNOS, *F*_training_ = 1.398, *F*_sex_ = 0.785, *F*_int_ = 0.029, df_training_ = 1, df_sex_ = 1, df_int_ = 1, *P*_training_ = 0.264, *P*_sex_ = 0.396 and *P*_int_ = 0.868; Anti-VEGFR-1, *F*_training_ = 1.99, *F*_sex_ = 0.00448, *F*_int_ = 0.118, df_training_ = 1, df_sex_ = 1, df_int_ = 1, *P*_training_ = 0.18, *P*_sex_ = 0.948 and *P*_int_ = 0.737

In the performed immunohistochemical studies measuring oxidative and nitrative stress (HNE, PAR, NT and eNOS markers) we did not find a significant difference between the trained and control groups, nor was there any gender difference in these markers (Fig. 7). Furthermore, no differences were found in the staining of VEGFR-1 receptors (Fig. 7).

**Fig. 8:**
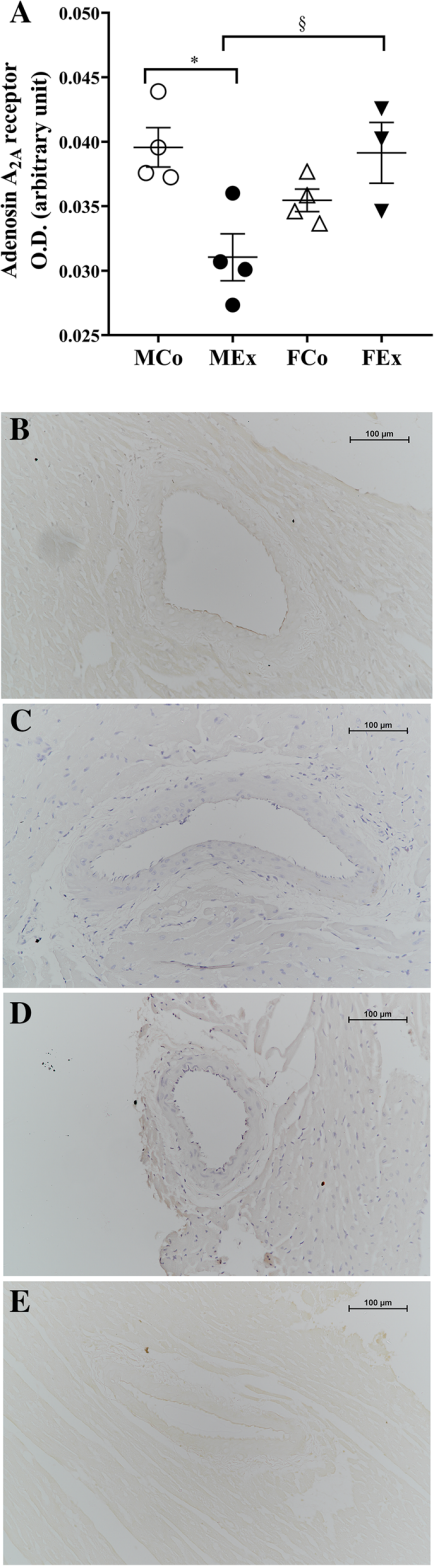
Results of AdenosineA_2A_ receptor (AdeA_2A_-R) immunohistochemical staining. **a** AdeA_2A_-R optical density (*n* = 4–4–4–3); **b** representative images of AdeA_2A_-R stained arteriole segments from MSed, MEx, FSed and FEx groups. Scale bar, 100 μm. Two-way ANOVA with post hoc Tukey’s test. (*F*_training_ = 2.166, *F*_sex_ = 1.465, *F*_int_ = 13.779, df_training_ = 1, df_sex_ = 1, df_int_ = 1, *P*_training_ = 0.169, *P*_sex_ = 0.251 and *P*_int_ = 0.003)

AdenosineA_2A_ receptor intensity was significantly decreased in males during exercise compared with the control group. Furthermore, the AdeA_2A_ receptor intensity of trained males was significantly lower even compared to female swimmers. In females, there was no change in AdeA_2A_ receptor expression upon exercise (Fig. 8).

**Fig. 9:**
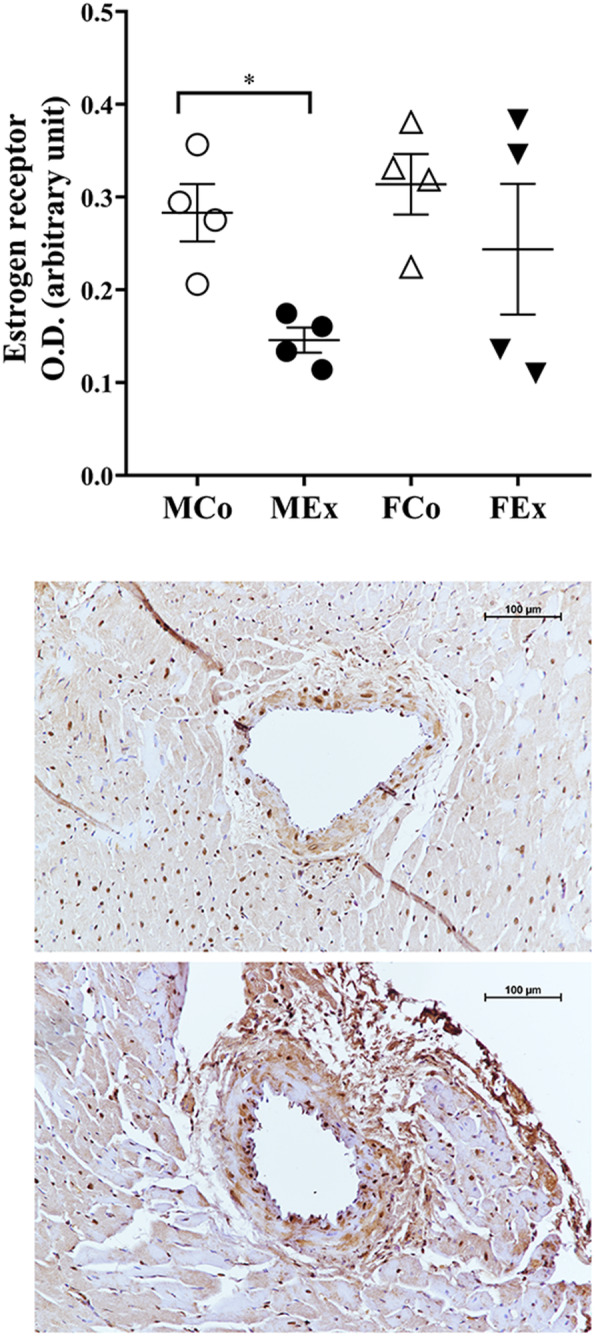
Results of estrogen receptor (ER) immunohistochemical staining. **a** ER optical density (*n* = 4–4–4–4); **b** representative images of ER stained arteriole segments from MSed, MEx, FSed and FEx groups. Scale bar, 100 μm. Two-way ANOVA with post hoc Tukey’s test. (*F*_training_ = 6.004, *F*_sex_ = 2.317, *F*_int_ = 0.633, df_training_ = 1, df_sex_ = 1, df_int_ = 1, *P*_training_ = 0.031, *P*_sex_ = 0.154 and *P*_int_ = 0.442)

Estrogen receptor intensity was significantly decreased in trained males compared with their control group (Fig. 9). It is possible that the estrogen receptor plays a role not only in sex differences in myocardial hypertrophy [49, 50] but also in sex differences in vascular adaptation.

## Discussion

In the present study, we first provide a reliable characterization of coronary resistance artery network remodeling in a rodent model of strenuous endurance training in which we applied an in situ micropreparation perfusion technique [7, 48]. In addition, we described the differences between males and females in both the sedentary and the trained states. Our results may be due to altered expression of estrogen receptor (ER) and AdeA_2A_ receptor.

### Physiological parameters

Numerous animal and human studies investigated the physiological left ventricular hypertrophy induced by exercise training [50–52]. In agreement with most of the studies in rodents [45, 49, 50], an increased relative LV mass (LV mass index) and systolic function (EF and FS) were observed in both male and female rats after our 12-week swim training programme. These alterations are a consequence of hypertrophy of ventricular myocytes [45]. Similar to our findings, Oláh et al. found that the body weight decreased only in the male exercised groups, whereas it did not change in the females [50]. Despite the left ventricular hypertrophy, the mean arterial pressure did not differ between the groups, which also illustrates the physiological nature of athletes’ hearts.

Female animals had lower body weights in both the sedentary and exercised groups, similar to earlier publications in rodents and humans [49, 53]. The LV mass index raised more in female animals than in males, in accordance with earlier studies [49, 50, 54, 55]. Estrogen receptor type beta might be responsible for this difference [49]. Furthermore, Oláh et al. found that the ERK1/2, S6 and mTOR activation levels differed in the hearts of swimming female and male rats [50]. In our case, the LV mass index also differed between the two sexes in the sedentary state, with females having higher values. Despite the sex differences in LV index, no sex differences could be identified regarding systolic function (FS and EF) in the exercised groups in our study.

### Coronary bifurcations (branchings)

No significant differences in the bifurcation geometry could be identified between either the sedentary and trained groups or the sexes. All four groups adhered fairly well to Murray’s law [56], which holds that the sum of the cubes of lumen radii of daughter branches should be equal to the cube of the lumen diameter of the mother branch (Fig. 2), ensuring that the stabilization of endothelial shear was effective in coronary networks formed during the sedentary and trained states in both sexes. The validity of Murray’s law was proven in coronary networks of aged rats [7] and in female rats made hypertensive by the chronic infusion of angiotensin II [6]. In all four groups, we found a fairly negative correlation between mother and daughter lumen ratios and angles of the daughter branch with the axis of the mother branch: while larger branches tended to follow the course of the mother branch, smaller branches deviated from it more (Fig. 3). Despite substantial left ventricular mass elevation, no branching deformities developed, similar to those found in aged [7] and in hypertensive [6] networks.

### Wall thickness

As described above, whole networks were (theoretically) divided into 50-μm ring units, with 29,390 such “ring” units for the 38 animals. Fig. 4a demonstrates that as an effect of training, wall thickness significantly increased in the 200- to 500-μm outer diameter range in males. We think that increased muscularity ensured increased range of vasomotion (between fully contracted and fully relaxed states), as has been observed in an earlier publication from our laboratory [25] with pressure arteriography for rat coronary arteriole segments in the 200-μm range. There was some sex significance in this respect: training-induced wall thickening was moderate in female (300–350 μm) vessels, and there was a characteristic thinning of the wall at the largest branches in this sex (Fig. 4b). There was no consensus in the literature as to the effect of training on coronary artery wall thickness: different parts of the circulation may be affected in a different manner [16, 21, 57, 58].

### Frequency of ring units building up the network

Exercise-induced remodeling of the coronary resistance artery system can be spotted on the frequency diagrams of the ring units forming the whole network. In males, the number of 400-μm units almost doubled, and there was also a substantial elevation in the 200-μm range (Fig. 5a). A similar alteration could be observed in females, but in the 150-μm diameter range. However, in females, the 350-μm units substantially reduced in their number (Fig. 5b). As there was no alteration in the ring elements themselves, we have good reason to think that morphological dilation of earlier existing thinner vessels occurred. This result is not surprising because the elevated myocardial mass requires increased perfusion, which can be provided by a remodelled coronary circulation [59]. As a result of endurance exercise, the cardiac performance improves in healthy subjects, and the tolerance for ischaemia and reperfusion injury increases even in patients with LV dysfunction [60–62]. The decreased risk of ischemic heart disease might be due to increased capillary density and neo-angiogenesis in cardiac mass as a result of exercise [39, 63]. Such events could have occurred in our case also, but these smaller resistance vessels penetrating the ventricular wall perpendicularly were outside the scope of our video microscopic study.

### Statistical geometry of the sedentary and exercised networks

Despite the above-mentioned limitations, our technique yields a further possibility to pinpoint at what points of the network these remodeling processes have taken place. An analysis of the two-dimensional flow distance–diameter ring unit frequency plots shows that in males, the elevation of 400- and 200-μm units occurred at just the right place: very close—at 2 mm from the orifice for the thicker, 400-μm vessels, and farther from the orifice (8–12 mm) for the 200-μm ones (Fig. 6a and c). As a result, the exercised network became younger [7] and healthier [6]. There was a clear, hemodynamically advantageous tendency: thicker vessels were closer and thinner ones were farther from the orifice. We can observe a substantially different situation in females: in their case, the sedentary pattern seems to be the more coherent (thinner vessels being farther from the orifice—see the oblique line dominating the diagram of Fig. 6b). In the network developed under the effect of strenuous exercise, the newly appearing population of 200- to 250-μm vessels appeared at not the ‘right’ place, far from the orifice, though at a distance of 5–8 mm, destroying the hemodynamically advantageous oblique pattern.

### Sex differences found

As described above, we found substantial sex differences in the coronary network in the control groups and in the swim-trained animals. The effect of this strenuous training was also different for the two sexes. The exercise load we applied was similar for males and females. Nevertheless, in females, the elevation of body mass practically ceased, and the improvement of ventricular function was more moderate than in the case of males. Increased muscularity (wall thickness) of the coronary vessels was missing; in effect, there was a thinning of the walls of larger vessels. The remodelling of the network seemed to be less advantageous than in exercised males.

There are substantial differences in the coronary epidemiology between men and women, especially before female menopause [64, 65], when the reduction in estrogen levels has a disadvantageous cardiovascular effect [66, 67]. The mechanism of this needs further explanation. Estrogen ß receptor (ERß) stimulation increased coronary angiogenesis even in male mice suffering from heart failure [43], and a loss of ERß activity increased coronary risk [43, 68–72]. Estrogen stimulates angiogenesis in several tissues through increasing the expression of VEGF, bFGF and eNOS [43, 73, 74] and improves endothelial dilation [44, 75, 76].

### Immunohistochemical examinations

The imbalance between oxidative and nitrative stress, and antioxidant protection damages cells and tissues at many points. In many conditions and diseases, this balance is significantly disturbed and shifted in the direction of oxidative stress. All of these are the causes or aggravating factors of various diseases, such as inflammatory conditions, cardiovascular diseases or cancerous lesions [77]. Regular physical exercise generally reduces chronic oxidative stress [30]. In our present work, with regards to the degree of oxidative and nitrative stress we found no difference between males and females, or between trained and control groups. This may be explained by the fact that the mechanisms responsible for antioxidant protection may become more prominent as a result of long-term training [30, 78].

Interestingly, we found no difference in the staining of VEGFR-1. It has been previously reported in the literature that VEGF mRNA levels increase in striated muscle and myocardium as a result of exercise [39]. However, the literature data on VEGF are not consistent; Kraus et al. found that although acute training increases VEGF in trained men, long-term training does not change circulating VEGF levels at rest [79], there was no difference between trained and untrained, or between men and women with respect to circulating VEGF levels [79, 80]. The opposite results may be due to the fact that VEGF may play a role in angiogenesis at the beginning of training, but is not involved in the maintenance of capillary network during long-term exercise [81].

According to the results of adenosine A_2A_ receptor staining, we found that training in males reduced the intensity of staining compared with both control males and trained females. Adenosine A_2A_ receptor expression is increased in hypoxic and ischemic conditions [82, 83]. This fact, and the fact that in our swim-trained animals, on the contrary, the receptor intensity decreased suggests that long-term training improved the myocardial oxygen supply in males.

In our research, we examined the intensity of estrogen receptors by immunohistochemical methods. As a result of training, the intensity of estrogen receptor staining decreased in males.

The potential clinical significance of network pattern for sport adaptation is that geometry of the resistance artery network determines whether the blood supply ventricular tissue follows homogenous pattern, and increased metabolic needs all part of the tissue is properly supplied with oxygenised blood.

### Perspectives and significance

We have provided the first targeted analysis of the long-term exercise adaptation of the resistance-sized coronary artery network geometry as it depends on sex. Wall thickness increases in males. The number of 400- and 200-μm units increased in males, while in females, narrowing of larger (350 μm) vessels and appearing of a new rich, 200–250-μm population close to the orifice, were characteristic.

## Conclusions

Our experiments have proven that, parallel with improvements in ventricular function, substantial remodelling of the coronary resistance artery network geometry occurs in the hearts of rats subjected to a strenuous, lasting swim exercise programme. There is a substantial sex difference in this process: in males, wall thickness increases, with a morphological dilation of vessels, increasing the numbers of 400- and 200-μm units, while in females, a narrowing of larger (350 μm) vessels and the appearance of a new, rich, 200- to 250-μm population close to the orifice are characteristic.

## Data Availability

All data generated or analysed during this study are included in this published article (and its supplementary information files).

## Abbreviations

AdeA_2A_: Adenosine A_2A_ receptor
AWTd: Anterior wall thickness in diastole
EF: Ejection fraction
eNOS: Endothelial nitric oxide synthase
ER: Estrogen receptor
FEx: Female exercised
FS: Fractional shortening
FSed: Female sedentary
HNE: 4-Hydroxy-2-nonenal
LAD: Left anterior descending
LV: Left ventricle
LVEDD: LV end-diastolic diameter
LVESD: LV end-systolic diameter
MEx: Male exercised
MSed: Male sedentary
nKR: Normal Krebs-Ringer solution
NT: 3-Nitrotyrosine
PAR: **P**oly(ADP)-ribose
PWTd: Posterior wall thickness in diastole
VEGFR-1: Vascular endothelial growth factor receptor 1
