# Correction to: Network analysis of the left anterior descending coronary arteries in swim-trained rats by an in situ video microscopic technique

**DOI:** 10.1186/s13293-021-00385-0

**Published:** 2021-06-18

**Authors:** Marianna Török, Petra Merkely, Anna Monori-Kiss, Eszter Mária Horváth, Réka Eszter Sziva, Borbála Péterffy, Attila Jósvai, Alex Ali Sayour, Attila Oláh, Tamás Radovits, Béla Merkely, Nándor Ács, György László Nádasy, Szabolcs Várbíró

**Affiliations:** 1 Department of Obstetrics and Gynecology, Semmelweis University, Üllői u. 78/a, Budapest, 1082 Hungary; 2 Institute of Clinical Experimental Research, Semmelweis University, Tűzoltó u. 37-47, Budapest, 1094 Hungary; 3 Department of Physiology, Semmelweis University, Tűzoltó u. 37-47, Budapest, 1094 Hungary; 4 Department of Neurosurgery, Military Hospital, Róbert Károly körút 44, Budapest, 1134 Hungary; 5 Heart and Vascular Center, Semmelweis University, Városmajor u. 68, Budapest, 1122 Hungary

**Correction to: Biol Sex Differ 12, 37 (2021)**

**https://doi.org/10.1186/s13293-021-00379-y**

Following publication of the original article [1], the authors reported an error in Fig. 9. The corrected Fig. 9 is given below and the original article [1] has been corrected.

**Fig. 9:**
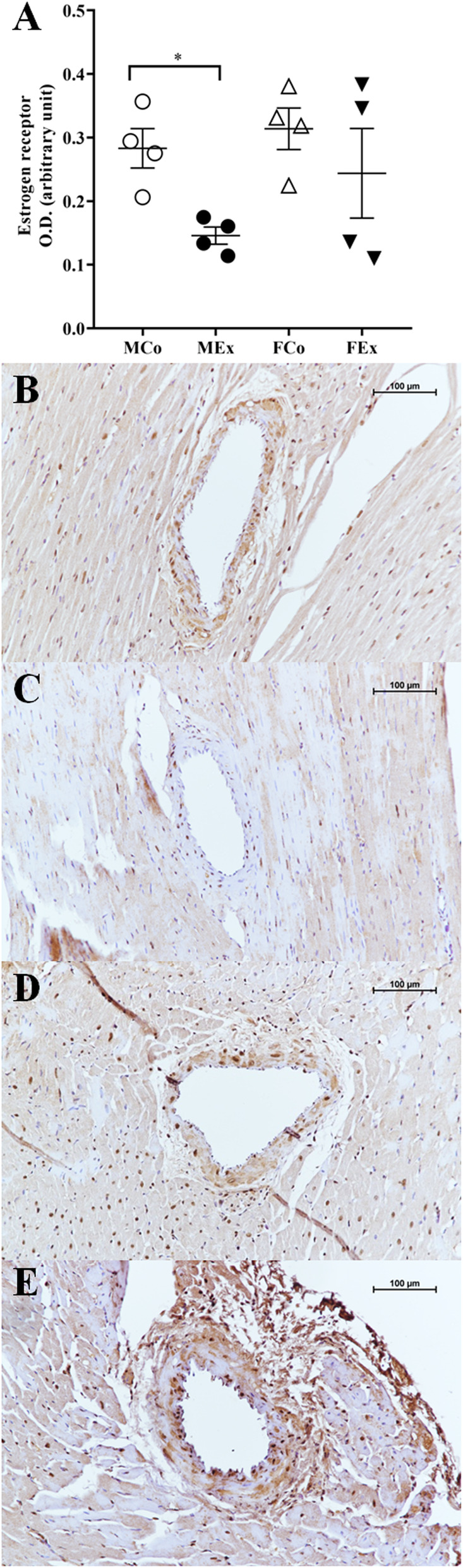
Results of estrogen receptor (ER) immunohistochemical staining. **a** ER optical density (*n* = 4–4–4–4); **b-e** representative images of ER stained arteriole segments from MSed, MEx, FSed and FEx groups. Scale bar, 100 μm. Two-way ANOVA with post hoc Tukey’s test. (F_training_ = 6.004, F_sex_ = 2.317, F_int_ = 0.633, df_training_ = 1, df_sex_ = 1, df_int_ = 1, P_training_ = 0.031, P_sex_ = 0.154 and P_int_ = 0.442)
